# Medical/Surgical, Cloth and FFP/(K)N95 Masks: Unmasking Preference, SARS-CoV-2 Transmissibility and Respiratory Side Effects

**DOI:** 10.3390/jpm12030325

**Published:** 2022-02-22

**Authors:** Dimitra S. Mouliou, Ioannis Pantazopoulos, Konstantinos I. Gourgoulianis

**Affiliations:** 1 Department of Respiratory Medicine, Faculty of Medicine, University of Thessaly, BIOPOLIS, 41110 Larissa, Greece; pantazopoulosioannis@yahoo.com (I.P.); kgourg@uth.gr (K.I.G.); 2 Department of Emergency Medicine, Faculty of Medicine, University of Thessaly, BIOPOLIS, 41110 Larissa, Greece

**Keywords:** SARS-CoV-2, transmission, masks, medical masks, FFP masks, N95 masks, cloth masks, respiratory side effects, cough, dyspnea, sputum

## Abstract

Background: Social distancing and mask-wearing were recommended and mandatory for people during the COVID-19 pandemic. Methods: A web-based questionnaire was disseminated through social media assessing mask type preference and COVID-19 history amongst tertiary sector services and the rates of the triad of respiratory symptoms in each mask type, along with other respiratory-related parameters. Results: Amongst 4107 participants, 63.4% of the responders, mainly women, preferred medical/surgical masks; 20.5%, mainly men, preferred cotton cloth masks; and 13.8% preferred FFP/(K)N95 masks. COVID-19 history was less common in FFP/(K)N95 compared to medical/surgical (9.2% vs. 15.6%, *p* < 0.001) or cloth masks (9.2% vs. 14.4%, *p* = 0.006). Compared to the control group (rare mask-wearing, nonsmokers and without lung conditions), those wearing one medical mask were more likely to report frequent sputum production (4.4% vs. 1.9%, *p* = 0.026) and frequent cough (4.4% vs. 1.6%, *p* = 0.013), and those wearing FFP/(K)N95 masks were more likely to report frequent cough (4.1% vs. 1.6%, *p* = 0.048). Compared to the control group, those preferring cotton cloth masks were more likely to report a frequent cough (7.3% vs. 1.6%, *p* = 0.0002), sputum production (6.3% vs. 1.9%, *p* = 0.003) and dyspnea (8% vs. 1.3%, *p* = 0.00001). Conclusions: Safe mask-wearing should be in parallel with a more personalized and social interaction approach.

## 1. Introduction

Coronaviruses have globally affected populaces since the early beginning of the 21st century. In December 2019, the novel severe acute respiratory syndrome coronavirus 2 (SARS-CoV-2) was identified from a cluster of cases of pneumonia in Wuhan, China [1]. On 30 January 2020, the World Health Organization (WHO) announced the coronavirus disease 19 (COVID-19) as a Public Health Emergency of International Concern, and a month and a half later, the COVID-19 epidemic was portrayed as a pandemic [2]. In the following two years, it seems that societies have acculturated SARS-CoV-2 and its mutants and that COVID-19 is likely to become an endemic disease.

Heretofore, scientific communities have made multifarious endeavors to monitor SARS-CoV-2 spread and to manage the COVID-19 pandemic. In particular, rapid testing, performed by qualified personnel, experts or even for self-diagnosis purposes, has been prevalent in populaces the last year, despite the fact that no method is completely foolproof [3,4]. Undeniably, the risk factors for a likely severe COVID-19 are prevalent, and, therefore, prevention against SARS-CoV-2 infection is highly required, especially for vulnerable cases, whereas vaccination strategies have been implemented for over a year now [5,6]. The WHO has recommended several ways for people to be protected against COVID-19, including vaccination, physical distancing, self-isolation of SARS-CoV-2 identified carriers, hand washing and the use of masks when physical distancing is not possible and in poorly ventilated settings [7].

Doubtlessly, masks are a key measure to suppress viral transmission and save lives, and depending on the type, masks can be used for either protection of healthy persons or to prevent onward transmission [8]. The WHO has also recommended the usage of medical masks predominantly for healthcare workers in clinical settings, symptomatic people, confirmed SARS-CoV-2 carriers, people with close contacts with COVID-19 cases, people over 60 and people with preexisting medical conditions that could place them at a risk for a likely severe COVID-19, whereas nonmedical masks can be used by the public under 60 and without underlying medical conditions [8].

Literature data also support that there is an association between mask use and SARS-CoV-2 transmission and that wearing a mask could reduce the risk of the infection [9,10]. Community mask use by healthy people could be beneficial, particularly for SARS-CoV-2, since transmission may occur in presymptomatic stage [11]. Moreover, given the current shortages of medical masks, the adoption of the public wearing of cloth masks is also recommended as an effective form of source control, in conjunction with existing hygiene, distancing and contact tracing strategies [12]. A review has stated that despite the lower efficiency of cloth masks compared to medical masks, laboratory results may underestimate the efficiency of cloth masks in real life [13]. A prepandemic article cautioned against the use of cloth masks in healthcare workers, since they may have an increased risk of infection [14]. However, the extended mask-wearing by the general population could lead to adverse effects and consequences in many medical fields [15].

The aim of this study is to present the mask type preferences amongst tertiary sector services and to monitor SARS-CoV-2 transmissibility in the wearing of specific mask types. Furthermore, the presence of the basic triad of respiratory symptoms is assessed for potential side effects in each mask type, and, finally, some future directions and aspects regarding future mask-wearing are well discussed.

## 2. Materials and Methods

### 2.1. WBQ Design

Although WBQs are currently being considered as a fluid form of observational, descriptive and analytical studies, they bespeak an upcoming propitious tool, enabling experts to combine ontological, ethical and epistemological principles to surveil society. WBQs enable motivated individuals to provide their answers, rapidly, at the touch of a button; they are automated, cost-effective and error-free [5,6,7]. The traditional closed-ended WBQs, structured with qualitative categorical or dichotomous questions, seem to be advantageous psychometric attempts and are desirable options for participation, contrary to open-ended questions requiring written answers [5,6,7].

Primarily, the WBQ of this study consisted of a binary question regarding gender. The occupation-related question was based on the sectors of classical economy, with further analysis in the tertiary sector, and included the following subgroups: (i) primary sector; (ii) secondary sector; (iii) tertiary sector with further subgrouping in public/private services, healthcare providers, food services, education, uniformed/military/policemen, freelancers and some other extra subgroups (for retirees, unemployed and university students). Considering the qualitative WBQ type for a better e-sample response, in addition to the fact that European countries are mainly aging, the concept of age-related questions was to follow a generation-based model with age ranges to reveal each generation’s criticism and attitudes. Generation categories included (i) Generation A, (ii) Baby Boomers, (iii) Generation X, (iv) Millennials, (v) Generation Z restricted in adults and (vi) Silent Generation (all labeled with age ranges as seen in 2021, i.e., <18, 18–24, 25–40, 41–56, 57–75, >75) [5,6,7]. Unemployed and retirees were included in the survey.

The exclusion criteria for the survey, regarding the parameter of age, were those aged under 18 and over 75. Furthermore, the study was solely designed for the tertiary sector services where social interactions are required, as aerosols and droplets may be controlled naturally in the primary sector and secondary sector industries are closed structures. Moreover, in the primary and secondary sectors, respiratory symptoms could be present due to inhalation of dust, particulate matters or heavy metals, and thus, respiratory symptom monitoring would not be precise.

Two questions regarding the frequency of mask-wearing were included, one for days per week and the other for hours per day, and individuals with at least 3 h of continuous daily mask use were analyzed for the third part of the survey since it is more accurate to monitor SARS-CoV-2 transmissibility for each mask type amongst those with frequent daily mask use rather than in those with spontaneous mask use. Another categorical question was included for the mask type, referring to (i) medical/surgical mask, (ii) two medical/surgical masks, (iii) FFP/(K)N95, (iv) cotton cloth masks and (v) other type cloth masks. Questions were also included for the frequency of cough, dyspnea and sputum, with three answers, (i) rarely, (ii) middle and (iii) frequently, and the last responses were considered for the study.

Apart from these basic questions, some other binary questions regarding COVID-19 status, smoking and chronic lung disease (CLD) status were included. The questionnaire also included questions regarding home geographical location, as well as outdoor and indoor air pollution, with the last one referring to fireplace/indoor pet since, generally, all people living in a house are somewhat exposed to indoor cleaning chemicals and kitchen pollution.

The control group of the fourth part of this study was the responders with rare nondaily mask use for no more than half an hour, nonsmokers and without CLD, whereas the mask-wearing group was those with at least 3 h of continuous daily mask use, with no smoking or CLD status, so as to be more accurate and precise with mask side effects.

### 2.2. Population-Based Sample and WBQ Administration

The survey was conducted in the Greek mainland, where strict lockdown policies were imposed the previous year and strict social distancing and mask use are presently implemented. Greece has also imposed strict limitations on nonvaccinated people in parallel with the ongoing financial crisis that the country is facing. The WBQ was disseminated around late November (18–27 November 2021), and adults were randomly invited to participate in the survey through social media shares in profiles and Facebook teams. Informed consent was obtained from all subjects during accepting participation in the study. WBQs were submitted in Google forms, and data were saved in an Excel spreadsheet.

### 2.3. Statistical Analysis

Statistical analyses were effectuated via the statistical software IBM SPSS Statistics for Windows, Version 26.0 (headquartered in Chicago). Data normality was assessed with Kolmogorov–Smirnov test. Tests were two-tailed, and the level of statistical significance was established at *p* ≤ 0.05. Chi-square test was applied for comparisons of frequencies, and Bonferroni correction was used for comparisons between subgroups. Spearman or Pearson/phi coefficients were used to evaluate correlations between variables.

## 3. Results

### 3.1. The Distribution of Genders and Generations by Tertiary Sector Services

The population-based sample consisted of 4107 participants, including 1129 (27.5%) men and 2978 (72.5%) women. Generation Z consisted of 623 (15.2%) participants, Millennials were about 2383 (58%) individuals, Generation X included 1000 (24.3%) individuals and 101 (2.5%) of the population-based sample were Baby Boomers. Table 1 illustrates the distribution of the responders in each service amongst genders and generations.

### 3.2. The Mask Types and Preference amongst Service Subgroups

In the whole population-based sample, 63.4% of the responders reported using medical/surgical masks, 20.5% reported wearing a cotton cloth mask and 13.8% reported a preference for FFP/(K)N95 masks. Women were more likely to prefer medical masks (65.5% vs. 57.8%, *p* < 0.05) while men were more likely to prefer cloth masks (29.4% vs. 20.4%, *p* < 0.05). Cotton cloth masks were mostly reported amongst Millennials (57.3%) and Generation X (28.9%), and medical/surgical masks were highly reported in the youth. Table 2 demonstrates the use of each mask type by job.

### 3.3. Mask Preference and SARS-CoV-2 Infection

Of the population-based sample, 80.4% reported daily mask-wearing for at least 3 h, and also 14.4% of them reported they had passed COVID-19. Amongst the responders with a frequent mask-wearing but who disclosed a history of SARS-CoV-2 infection, there was a significant difference for the FFP/(K)N95 masks compared to one medical/surgical mask (9.2% vs. 15.6%, *p* < 0.001) or cloth masks (9.2% vs. 14.4%, *p* = 0.006), whereas there was no significant difference for those reported the use of two medical/surgical masks (9.2% vs. 11.9%, *p* = 0.378). Table 3 shows the SARS-CoV-2 infection history in each job among each mask subtype.

### 3.4. Masks and Respiratory Side Effects

Of the responders, 45.8% reported being smokers and 8.6% reported a CLD status, and they were excluded from this part of the study. Thus, the control group consisted of 375 responders and the mask-wearing group consisted of 1673 responders, of whom 58.1% reported a preference for wearing one medical/surgical mask, 18% reported a preference for FFP/(K)N95 and 19% reported a preference for wearing a cotton cloth mask. Compared to the control group, those wearing one medical mask were more likely to report frequent sputum production (4.4% vs. 1.9%, *p* = 0.026) and frequent cough (4.4% vs. 1.6%, *p* = 0.013), but dyspnea showed no significant difference (3.1% vs. 1.3%, *p* = 0.069). Compared to the control group, those wearing FFP/(K)N95 masks were more likely to report frequent cough (4.1% vs. 1.6%, *p* = 0.048), while dyspnea and sputum production had no significant difference (2.4% vs. 1.3%, *p* = 0.308, and 2% vs. 1.9%, *p* = 0.866). Compared to the control group, those preferring cotton cloth masks were more likely to report a frequent cough (7.3% vs. 1.6%, *p* = 0.0002), sputum production (6.3% vs. 1.9%, *p* = 0.003) and dyspnea (8% vs. 1.3%, *p* = 0.00001). 

Generally, no significant differences were observed for genders’ respiratory symptoms and each mask type, except for cough and dyspnea that were absent in men preferring FFP(K)N95 masks. In addition, the younger generations were more likely to report respiratory symptoms compared to the older ones. Additionally, smokers showed higher rates of respiratory symptoms but showed the same variations as the mask-wearing group for each mask type, and responders with CLDs were more likely to report cough and dyspnea when wearing one medical/surgical mask rather than FFP/(K)N95 masks.

## 4. Discussion

In our study, more than half of the responders reported a preference for medical/surgical masks and one-fifth reported FFP/(K)N95 mask-wearing, and healthcare professionals highly shaped this rate. Medical/surgical masks were mostly preferred by women and youth, and healthcare providers showed the highest rate in wearing two medical/surgical masks, whereas mainly men, half of the Millennials, uniformed and unemployed preferred cotton cloth masks. The overall history of SARS-CoV-2 infection was less common amongst those with daily FFP/(K)N95 mask-wearing, but public education and food services showed the highest rates of infection compared to other tertiary sector services with that type of daily mask-wearing. The highest rates of SARS-CoV-2 infection were seen for cloth masks in healthcare providers, uniformed and university students, and, regarding medical/surgical masks, high rates were observed especially in food services and uniformed. Regarding respiratory side effects, the FFP/(K)N95 mask-wearing group was free of frequent sputum production and dyspnea compared to the control group, but frequent cough was statistically significantly more prevalent in this group than the control group but without any difference compared to medical/surgical masks, while frequent dyspnea showed no difference but frequent sputum and cough were significant compared to those with rare mask-wearing. Cotton cloth mask-wearing showed the highest percentages in all the analyzed respiratory symptoms, being significant compared to those without frequent mask-wearing and even amongst the other mask types. As expected, smokers showed higher rates in frequencies of all respiratory symptoms, but frequent cough and dyspnea in people with CLDs were more common in medical/surgical masks rather than FFP(K)N95 masks; thus, this mask type may be appropriate specifically for those with lung conditions.

On the whole, FFP/(K)N95 mask-wearing responders were significantly less likely to have a COVID-19 history. Women were more likely to prefer medical/surgical masks; they are more sensitive than men in health issues, and men mostly preferred cotton cloth masks. The WHO has recommended that healthcare providers should wear medical and FFP/(K)N95 masks, but, on the contrary, we revealed that some healthcare providers prefer cloth mask-wearing [8]. Doubtlessly, this fact is unacceptable for the health field during the COVID-19 pandemic. Fortunately, two medical/surgical mask-wearing was mostly seen in healthcare services. Sadly, a study revealed that less than half of healthcare professionals were informed about mask types against SARS-CoV-2 [16]. Doubtlessly, the percentage of penetration in cloth masks is higher than that in surgical masks or N95 respirators [17]. In our study, healthcare providers that are highly exposed to SARS-CoV-2 carriers were more likely to report a COVID-19 history with cloth mask-wearing, and public education teachers were more likely to catch the virus with cloth mask-wearing; teachers are in schools with children that easily transmit the virus and can pass it with mild symptoms without understanding it. In addition, university students showed high rates of COVID-19 history with cloth mask types since, undeniably, close contacts in the youth cannot be fully amended. Most uniformed work in closed structures in which it is easy for the virus to be transmitted, and they preferred cloth masks; unemployed responders preferred them too, which may be due to the cost compared to the others and the ability to wash and reuse them—a method being cost-free. Food services also showed high rates in cloth masks, but it is easy for delivery workers to catch and transmit the virus by contacting many people daily, and maybe that is why both medical/surgical and cloth mask-wearers reported high rates of COVID-19 history. Despite the fact that uniformed can transmit the virus in their closed structures, partially explaining their higher rates of COVID-19 history, another study revealed that those working in food services were more vulnerable to SARS-CoV-2 infection [18]. The WHO also recommended people with health risks to be well protected, but in our study, smokers and people with CLDs showed various preferences for mask types, even cloth masks. Regarding freelancers, there was no difference for COVID-19 history in various mask types, but some of their work includes vigorous physical activity, and it should be further discussed to what extent should they wear a mask during work since the WHO recommended that even in an area of SARS-CoV-2 transmission masks should not be worn because of the risk of reducing breathing capacity [8]. However, generally speaking, the efficacy of medical masks is not the same as that of cloth masks for respiratory viral transmission [19]. Since a sole cloth fabric is not a material designed solely to be a face mask and protect against pathogen transmission, we highlight the need for cloth masks to be disallowed in specific services.

To our knowledge, this is the first study to evaluate the triad of respiratory symptoms amongst the wearing of various mask types. In our study, the daily medical/surgical mask-wearing was more likely to show frequent sputum production and cough in comparison with rare/spontaneous mask-wearing. Several effects of mask-wearing have been discussed, such as physiological adverse effects in cardiopulmonary exercise capacity, including increased rebreathing of expelled carbon dioxide, significant increased respiratory rate, hyperventilation, increase in CO_2_ in the blood, hypoxemia and hypercapnia [20]. Cotton cloth mask-wearing showed the highest rates in the triad of respiratory symptoms in our population-based sample. However, no further nonrespiratory symptoms were evaluated in this study, and another study revealed adverse skin reactions due to medical masks compared to cloth masks [21]. More targeted studies, in the future, should analyze the possibility of the prolonged wearing of cotton cloth masks leading to early byssinosis signs, since it is an evident lung condition among cotton workers due to fiber inhalation [22]. Nevertheless, cotton cloth masks not only were not such effective in preventing SARS-CoV-2 transmission, but also had the highest levels of respiratory side effects; additionally, daily FFP/(K)N95 mask-wearing responders were less likely to have COVID-19 history and also had lower levels of respiratory side effects. Frequent dyspnea and sputum production were not significantly seen in FFP/(K)N95 mask-wearing, and cough rates (only women reported cough) were not much different from those seen in medical/surgical mask-wearing, yet significantly different compared to the control group. Even if cough was the only respiratory symptom seen in this mask type, a study studying healthcare professionals showed that prolonged use of medical and N95 masks had caused headaches, rash, acne, skin breakdown and impaired cognition in most of those surveyed [23]. The authors suggest frequent breaks, improved hydration and rest and skin care for healthcare professionals with prolonged mask-wearing [23]. We also highlight the need for FFP/(K)N95 masks to be thoroughly studied for other potential adverse effects since, in this study, we have assessed only some basic respiratory issues.

Further studies are needed to finally evaluate if mask-wearing is effective or if the effectiveness is attributable to the social distancing and other personal care and protection strategies and the overall psychology amongst people, as several side effects of mask-wearing have been reported in current literature. Some variations in COVID-19 history amongst healthcare professionals, unemployed or food services—comparing rates of infection of each mask type—could show that in specific services where viral transmission is high, mask type efficacy may not be efficiently monitored, or that social distancing and personalized protection strategies may play a more important role in preventing transmission. Public health strategies may have overreacted in this pandemic, and the medical motto “primum non nocere” (“first, do no harm”), a moral principle everyone should at least consider following, was evidently not observed during the pandemic [20]. Moreover, presymptomatic carriers may transmit the virus, but false positives are evident, and a current perspective doubted the realistic existence of asymptomatic patients in COVID-19 [3,24]. As a result, maybe it is healthier for symptomatic patients to wear masks so as not to transmit the virus to others and for all the others being at risk for a likely severe COVID-19 to wear them only in crowded places with high social interactions or in places with a high risk of SARS-CoV-2 transmission. However, vaccinated people should follow the same path since, even if they will likely pass COVID-19 with mild symptoms, they can still transmit the virus to others [6,25]. Finally, there may be a need for some more safe mask designs for future epidemics, and it is required that safe mask-wearing be acculturated in society, especially in environmental pollution such as during summer fires in some Mediterranean cities or even in extreme air pollution due to cars or because of fireplace smoke in winter.

No study is completely foolproof. Besides healthcare professionals to some extent, we do not know if all the others were equally exposed to the virus; in what conditions they caught the virus; and if it happened during work, on transportation, at a friend or family level of transmission or elsewhere. People started wearing N95 masks later due to limited availability at the beginning of the pandemic, and we do not know when the participants contracted the virus. However, respiratory side effects are irrelevant to this parameter, but we did not exclude those with allergies as no related question was included in our WBQ. In addition, the FFP/(K)N95 mask-wearing group could be larger so as to analyze more accurately respiratory symptoms and their potential respiratory safety.

## 5. Conclusions

FFP/(K)N95 mask-wearing responders were less likely to have a COVID-19 history and were less likely to report respiratory symptoms, compared to the other mask types. Cotton cloth masks not only did not prevent SARS-CoV-2 transmission but also were more likely to cause frequent cough, dyspnea and sputum production. Public health strategies may have overreacted during the pandemic; mask-wearing but with safe mask-types should follow a more personalized and social interaction approach, and safe mask-wearing should also be recommended in future epidemics or environmental issues.

## Figures and Tables

**Table 1:** Distribution of genders and generations by tertiary sector services.

Tertiary Sector Services	*n*	Gender		*p*-Value	Generations			
		Male (% out of *n*)	Female (% out of *n*)		Generation Z (% out of *n*)	Millennials (% out of *n*)	Generation X (% out of *n*)	Baby Boomers (% out of *n*)
Healthcare providers	381	65 (17.1)	316 (82.9)	<0.001	38 (10) ^a^	249 (65.4) ^b^	88 (23) ^a,b^	6 (1.6) ^a^
Food Services	300	93 (31)	207 (69)	0.157	66 (22) ^a^	188 (62.7) ^a,b^	45 (15) ^a,b^	1 (0.3)^c^
Public education	194	33 (17)	161 (83)	<0.001	10 (5.2) ^a^	113b (58.2) ^a^	65 (33.5) ^a,b^	6 (3.1) ^a^
Private education	188	23 (12.2)	165 (87.8)	<0.001	19 (10.1) ^a^	132 (70.2) ^b^	35 (18.6) ^a,b^	2 (1.1) ^a^
Uniformed	74	54 (73)	20 (27)	<0.001	5 (6.8) ^a^	42 (56.7) ^b^	27 (36.5) ^a,b^	-
Freelancers	363	140 (38.6)	223 (61.4)	<0.001	10 (2.8) ^a^	224 (61.7) ^b^	121 (33.3) ^b^	8 (2.2) ^a^
University students	399	107 (26.8)	292 (73.2)	<0.001	326 (81.7) ^a^	73 (18.3) ^b^	-	-
Other public services	222	66 (29.7)	156 (70.3)	0.442	14 (6.3) ^a^	87 (39.2) ^a,b^	111 (50) ^b^	10 (4.5) ^a^
Other private services	1570	467 (29.7)	1103 (70.3)	0.010	114 (7.3) ^a^	1057 (67.2) ^b^	384 (24.5) ^c^	15 (1) ^a^
***Retirees***	63	16 (25.4)	47 (74.6)	0.707	-	-	19 (30.1) ^a^	44 (69.9) ^b^
***Unemployed***	353	65 (18.4)	288 (81.6)	<0.001	21 (5.9) ^a^	219 (62) ^b^	104 (29.5) ^a,b^	9 (2.5) ^a^

* Each subscript letter denotes a subset of generation categories whose column proportions do not differ significantly from each other at the 0.05 level.

**Table 2:** Mask type preference/wearing by job.

Tertiary Sector Services	*n*	Mask Type				
		One Medical/Surgical Mask (% out of *n*)	Two Medical/Surgical Masks (% out of *n*)	FFP/(K)N95 Mask (% out of *n*)	Cotton Cloth Mask (% out of *n*)	Other Cloth Mask (% out of *n*)
Healthcare providers	381	243 (63.8) ^a^	28 (7.3) ^b^	82 (21.5) ^b^	25 (6.6) ^c^	3 (0.8) ^a,c^
Food services	300	188 (62.7) ^a^	4 (1.3) ^a,b^	14 (4.7) ^b^	76 (25.3) ^a^	18 (6) ^c^
Public education	194	108 (55.7) ^a^	6 (3.1) ^a^	41 (21.1) ^b^	37 (19.1) ^a,b^	2 (1)
Private education	188	108 (57.4) ^a^	7 (3.7) ^a^	34 (18.1) ^a^	34 (18.1) ^a^	5 (2.7) ^a^
Uniformed	74	33 (44.6) ^a^	1 (1.4) ^a,b^	8 (10.8) ^a,b^	27 (36.5) ^b^	5 (6.8) ^b^
Freelancers	363	216 (59.5) ^a^	6 (1.7) ^a^	60 (16.5) ^a^	68 (18.7) ^a^	13 (3.6) ^a^
University students	399	270 (67.7) ^a^	14 (3.5) ^a,b^	48 (12) ^a,b^	63 (15.8) ^b^	4 (1) ^a,b^
Other public services	222	135 (60.8) ^a^	9 (4.1) ^a^	31 (14) ^a^	43 (19.4) ^a^	4 (1.8) ^a^
Other private services	1570	949 (60.4) ^a^	36 (2.3) ^a^	201 (12.8) ^a^	351 (22.4) ^a^	33 (2.1) ^a^
***Retirees***	63	40 (63.5) ^a^	1 (1.6) ^a^	7 (11.1) ^a^	12 (19) ^a^	3 (4.8) ^a^
***Unemployed***	353	193 (54.7) ^a^	7 (2) ^a,b^	39 (11) ^a^	107 (30.3) ^b^	7 (2) ^a,b^

* Each subscript letter denotes a subset of mask categories whose column proportions do not differ significantly from each other at the 0.05 level.

**Table 3:** History of SARS-CoV-2 infection amongst services by mask type.

Tertiary Sector Services	*n*	History of SARS-CoV-2 Infection (% out of *n*)	Mask Type		
			Medical/Surgical Mask (%) *	FFP/(K)N95 Mask (%) *	Cloth Mask (%) *
Healthcare providers	353	42 (11.9)	28 (11.2) ^a^	9 (11) ^a^	5 (22.7) ^b^
Food services	251	48 (19.1)	34 (21.1) ^a^	2 (15.4) ^b^	12 (15.6) ^b^
Public education	181	33 (18)	20 (18.7) ^a^	8 (1.5) ^a^	5 (14.3) ^b^
Private education	177	22 (12.4)	14 (13) ^a^	2 (5.9) ^b^	6 (17.1) ^a^
Uniformed	52	12 (23.1)	5 (20.8) ^a^	-	7 (35) ^b^
Freelancers	251	29 (11.6)	17 (11.6) ^a^	6 (11.1) ^a^	6 (12) ^a^
University students	355	63 (17.7)	47 (18.8) ^a^	3 (6.4) ^b^	13 (22.4) ^c^
Other public services	199	27 (13.6)	19 (14.4) ^a^	2 (6.6) ^b^	6 (14.6) ^a^
Other private services	1301	173 (13.3)	128 (15.3) ^a^	14 (7.4) ^b^	31 (11.9) ^a^
***Retirees***	17	2 (11.8)	1 (9) ^a^	-	1 (50) ^b^
***Unemployed***	163	24 (14.7)	19 (19.2) ^a^	3 (10.3) ^b^	2 (5.7) ^c^

* Each subscript letter denotes a subset of mask type categories in each row whose column proportions do not differ significantly from each other at the 0.05 level, and percentages refer to the number of COVID-19 cases in each mask subgroup of each row.

## Data Availability

Data sharing is not applicable to this article. The data are not publicly available due to restrictions. They contain information that could compromise the privacy of the participants.
